# *Trypanosoma cruzi* Infection Is a Potent Risk Factor for Non-alcoholic Steatohepatitis Enhancing Local and Systemic Inflammation Associated with Strong Oxidative Stress and Metabolic Disorders

**DOI:** 10.1371/journal.pntd.0003464

**Published:** 2015-02-10

**Authors:** Luisina I. Onofrio, Alfredo R. Arocena, Augusto F. Paroli, María E. Cabalén, Marta C. Andrada, Roxana C. Cano, Susana Gea

**Affiliations:** 1 Centro de Investigaciones en Bioquímica Clínica e Inmunología CIBICI-CONICET, Facultad de Ciencias Químicas, Universidad Nacional de Córdoba, Córdoba, Argentina; 2 Facultad de Ciencias Químicas, UA Área CS.AGR.ING.BIO Y S-CONICET. Universidad Católica de Córdoba, Córdoba, Argentina; School of Population Health, University of Queensland, AUSTRALIA

## Abstract

**Background:**

The immune mechanisms underlying experimental non-alcoholic steatohepatitis (NASH), and more interestingly, the effect of *T. cruzi* chronic infection on the pathogenesis of this metabolic disorder are not completely understood.

**Methodology/Principal Findings:**

We evaluated immunological parameters in male C57BL/6 wild type and TLR4 deficient mice fed with a standard, low fat diet, LFD (3% fat) as control group, or a medium fat diet, MFD (14% fat) in order to induce NASH, or mice infected intraperitoneally with 100 blood-derived trypomastigotes of Tulahuen strain and also fed with LFD (I+LFD) or MFD (I+MFD) for 24 weeks. We demonstrated that MFD by itself was able to induce NASH in WT mice and that parasitic infection induced marked metabolic changes with reduction of body weight and steatosis revealed by histological studies. The I+MFD group also improved insulin resistance, demonstrated by homeostasis model assessment of insulin resistance (HOMA-IR) analysis; although parasitic infection increased the triglycerides and cholesterol plasma levels. In addition, hepatic M1 inflammatory macrophages and cytotoxic T cells showed intracellular inflammatory cytokines which were associated with high levels of IL6, IFNγ and IL17 plasmatic cytokines and CCL2 chemokine. These findings correlated with an increase in hepatic parasite load in I+MFD group demonstrated by qPCR assays. The recruitment of hepatic B lymphocytes, NK and dendritic cells was enhanced by MFD, and it was intensified by parasitic infection. These results were TLR4 signaling dependent. Flow cytometry and confocal microscopy analysis demonstrated that the reactive oxygen species and peroxinitrites produced by liver inflammatory leukocytes of MFD group were also exacerbated by parasitic infection in our NASH model.

**Conclusions:**

We highlight that a medium fat diet by itself is able to induce steatohepatitis. Our results also suggest a synergic effect between damage associated with molecular patterns generated during NASH and parasitic infection, revealing an intense cross-talk between metabolically active tissues, such as the liver, and the immune system. Thus, *T. cruzi* infection must be considered as an additional risk factor since exacerbates the inflammation and accelerates the development of hepatic injury.

## Author Summary

Chagas disease caused by the protozoan parasite *T*. *cruzi* is a neglected tropical disease widespread in Latin America, and non-alcoholic steatohepatitis constitutes a prominent health concern with increasing incidence of obesity and diabetes worldwide. Parasitic infection induced marked metabolic changes, improved insulin resistance, but it increased the triglycerides and cholesterol plasma levels. Our findings demonstrate for the first time a synergic effect between the hepatic damage caused during the steatohepatitis process (generated by a medium fat diet) and the exacerbated inflammation triggered by the chronic parasitic infection, revealing an intense cross-talk between the liver, a metabolically active tissue, and the immune system. We propose that *T*. *cruzi* infection must be considered as an additional risk factor for this metabolic disorder. These findings may provide new insight for the American Trypanosomiasis associated with the steatohepatitis.

## Introduction

The liver is a key organ that controls the metabolic homeostasis of lipids, carbohydrates, and proteins. Accumulative evidence demonstrates that the liver has specific immunological properties and contains a large number of resident and non-resident cells that participate in the regulation of inflammatory and immune responses [1,2]. The dysregulation of hepatic functions could lead to metabolic disorders. Society worldwide has experienced a lifestyle shift towards higher intake of fat food, chronic stress and less physical activity [3]. Fatty liver diseases are an increasingly common cause of liver disease that can progress to non-alcoholic steatohepatitis (NASH) and this constitutes a major health concern along with the increasing incidence of obesity and diabetes in the world. It is estimated that it affects at least 30% of the general population [4–7]. NASH is considered to be a chronic inflammatory liver disease influenced by risk factors of the metabolic syndrome [8] and by the insulin resistance (IR) associated with steatosis, overweight, dyslipidaemia, hyperinsulinaemia, and arterial hypertension [9]. But this process is not well understood. Inflammation is imposed on steatosis by recruitment and activation of inflammatory cells in the liver, which contributes to steatohepatitis [10]. This phenomenon can be explained by increased lipolysis from the fat cells or increased intake of dietary fat, followed by the enrichment of free fatty acids (FFA). In addition, *de novo* lipogenesis in the liver greatly contributes to hepatic steatosis. A cross-talk between different metabolically active tissues and the immune system has been also proposed [11].

In murine models of obesity induced by a high fat diet (HFD), macrophages switch to a M1-polarization state [12,13]. This phenomenon appears to be associated with C-C chemokine receptor 2 (CCR2)-dependent monocyte recruitment. Remarkably, the inhibition of MCP1/CCL2 induces an improvement of steatohepatitis and metabolic syndrome [14]. In addition, an imbalance of NK, DCs, B cells and different subtypes of T cells have been involved in steatohepatitis [15].

On the other hand, *Trypanosoma cruzi* is the protozoan parasite that causes Chagas disease, which is widespread in Latin America. It is estimated that 10 million people are infected worldwide and more than 25 million people are at risk of the infection [16]. However, in the past decades, mainly as a result of increased population migrations, the diagnosed cases have increased also in non-endemic countries such as Canada, United States of America and Europe [17]. Parasite persistence eventually results in severe complications in the cardiac and gastrointestinal tissues. *T*. *cruzi* also infects the reticuloendothelial system including liver, spleen, and bone marrow [18]. We have previously studied the innate immune response and liver damage triggered by *T*. *cruzi* (Tulauhen strain) during acute infection in mice [19].

Toll-like receptors (TLR) recognize pathogen-associated molecular patterns (PAMPs) but also bind damage-associated molecular patterns (DAMPs), which are molecules released by sterile injury. Thus, PAMPs and DAMPs that bind to the same type of receptor initiate similar intracellular pathways terminating in identical effector functions, which play a crucial role in the pathogen clearance as well as in the onset of steatohepatitis. Thus, different *T*. *cruzi* ligands for TLR2 and TLR4, among others, have been identified [20]. Moreover, MyD88 KO mice were more susceptible to the infection showing high parasitemia and mortality [21]. Our group demonstrated for the first time a differential modulation in the expression of TLR2, TLR4 and TLR9 in hepatocytes from infected mice. Furthermore, endogenous molecules produced by dying cells, or in certain pathological conditions, such as excessive FFA, denatured host DNA and oxidized low density lipoprotein (oxLDL), may stimulate TLRs resulting in the development or acceleration of inflammatory diseases [22,23]. Although chronic stimulation of the innate immune system is believed to underlie the pathology of these diseases, the molecular mechanisms of activation remain unclear. Genetic deficiency of TLR4 or MyD88 is associated with a significant reduction of hepatic injury in hypercholesterolemic mouse model, providing a pathophysiologic link between innate immunity, inflammation, and steatohepatitis [24–27]. CD36 scavenger receptor was also involved in the development and progression of esteatohepatitis [28] as well as in atherosclerosis [29] promoting sterile inflammation through assembly of a TLR4 and TLR6 heterodimer [30,31]. Even though there have been enough evidence of these disorders, the understanding of the effect of *T*. *cruzi* infection on the pathogenesis of these processes has been poorly explored [32].

In this work we focus on critical aspects of liver innate and adaptive immune cells, the TLR4 signaling, inflammatory cytokines and immune mechanisms underlying a chronic steatohepatitis experimental model and we also investigate how *T*. *cruzi* infection influences the evolution of this metabolic disorder. We emphasize that medium fat diet by itself is able to induce steatohepatitis, but interestingly, the combination with this parasite infection induces marked metabolic changes in the host, suggesting an intense synergism between the immune response provoked by DAMPs raised as a consequence of the steatohepatitis and PAMPs of *T*. *cruzi*. Our results demonstrates that an exacerbated inflammatory process induced by the infection could be detrimental for the steatohepatitis. Moreover, this process correlated with higher hepatic parasite load in I+MFD compared to the I+LFD group. These findings, may provide novel avenues for therapeutic targets of NASH, the most common chronic liver disease in the world associated with American Trypanosomiasis.

## Materials and Methods

### Ethic statement

All animal experiments were approved by and conducted in accordance with guidelines of the Committee for Animal Care and Use of the Facultad de Ciencias Químicas, Universidad Nacional de Córdoba (Approval Number HCD 388/11) in strict accordance with the recommendation of the Guide to the Care and Use of Experimental Animals published by the Canadian Council on Animal Care (OLAW Assurance number A5802–01).

### Mice and experimental design

C57BL/6J male mice were purchased from Facultad de Ciencias Veterinarias, Universidad Nacional de La Plata, Argentina and C57BL/10ScNJ mice lacking the Tlr4 gene (Tlr4lps-del mice) were from The Jackson Laboratory, Bar Harbor, ME, USA.

The mice were maintained under a standard light cycle (12 h light/dark) and were allowed free access to water and food. Six-to-eight-week-old male mice were fed with a standard low fat diet, LFD (3% from fat), or a medium fat diet, MFD (14% fat), or infected intra-peritoneally with 100 blood-derived trypomastigotes of Tulahuén strain and also fed with LFD (I+LFD) or MFD (I+MFD) for 24 weeks. LFD composition: 26% protein, 53% carbohydrate, 3% fat, 8% crude fiber and 10% total mineral; MFD composition: 25% protein, 50% carbohydrate, 14% fat, 3% crude fiber and 8% total mineral.

Infection was corroborated with the ELISA Chagas test (Wiener). Titles of IgG antibodies anti-*T*.*cruzi* higher than 1/1024 were detected at 24 weeks. Parasites were maintained by serial passages in mice. Parasitemia was monitored by counting the number of viable trypomastigotes in blood collected from the retrorbital sinus after lysis with a 0.87% ammonium chloride buffer. All animals were weighed, anesthetized with isofluorane and were sacrificed before liver tissue collection.

### Measurement of plasma lipids

After 4, 12 and 24 weeks, the mice were fasted for 10 hours, and blood samples were collected. Plasma total cholesterol (TC) and triglyceride (TG) concentrations were quantified with the use of enzymatic kits (Roche) in a Hitachi modular P800 autoanalyzer.

### Determination of homeostasis model assessment of insulin resistance

The homeostasis model assessment of insulin resistance (HOMA-IR) was calculated using the following formula: fasting blood glucose (mg/dl) × fasting insulin (μU/ml)/405. Plasma insulin levels were measured with an insulin RIA kit DPC Coat A Count (Siemens) using Ingetron MODEL MN2200-E. Plasma glucose levels were measured using an enzymatic kit (Roche) with Hitachi modular P800.

### Cytokine assays

The ELISA sandwich assay was performed for quantification of cytokine and chemokines levels. Briefly, ELISA plates were coated with anti-cytokine/chemokine antibodies (BD Pharmingen and e-Bioscience) overnight and then washed and blocked. The plasma obtained at 24 weeks was incubated overnight. After that, plates were subjected to biotinylated anti-cytokine/chemokine antibody (BD Pharmingen and e-Bioscience) for 1 h. After washing, the plates were incubated with streptavidin–peroxidase (BD Pharmingen), and washed once more. The reaction was revealed using TMB Substrate Chromogen (BD Pharmingen), before being read at 450 nm in a Microplate reader (Bio-Rad). Standard curves were generated using recombinant cytokines (BD Pharmingen and e-Bioscience).

### Histological analysis

Mice were euthanized and slowly perfused by intracardiac injection with 10 ml of heparin diluted in PBS after 10-h fast. Liver tissue was collected and samples were fixed with 10% PBS-formaldehyde and embedded in paraffin. Tissue sections were cut at a thickness of 5 μm and stained with hematoxylin and eosin (H&E). TG accumulation in the liver was examined by Sudan Black IV staining. Liver specimens were then evaluated by light microscopy using a Nikon Eclipse TE 2000 U equipped with a digital video camera.

### Measurement of TG and total cholesterol in the liver

Liver tissues were homogenized with PBS using a hand homogenizer. Homogenates were centrifuged at 5000 rpm for 10 min at 4°C. The supernatant was then collected and TG and cholesterol were determined using commercial enzymatic kits (Roche) in a Hitachi modular P800 auto-analyzer.

### Isolation of mouse intrahepatic leukocytes

Intrahepatic leukocytes (IHLs) were isolated from all mouse groups as previously described [33]. Briefly, liver in RMPI medium containing gentamicin (both from Gibco) plus 1% of FBS (Sigma-Aldrich) was passed through 100-mm nylon meshes; red blood cells were removed using lysis buffer (Sigma) and finally IHLs were obtained after 20 min centrifugation (600 × *g*) in a 35% and 70% bilayer Percoll (Sigma) gradient. Viable cells number was determined by Trypan blue exclusion.

### Flow cytometry

One million cells were washed in ice-cold FACS buffer (PBS-2% FBS) and incubated with fluorochrome labeled-Abs for 20 min at 4°C. Different combinations of the following Abs were used: 1) PE-labeled: anti- CD36, F4/80 or CD19; 2) APC or Alexa Fluor 647-labeled: anti- CD11c, NK1.1 or CD4; 3) PECy7-labeled: anti-F4/80; 4) PerCPCy5.5-labeled: anti- CD4 or CD3; 5) FITC-labeled: anti-CD3 or CD68; 6) biotin-labeled: anti-CD206 and SA-PEcy5. Intracellular cytokines were detected after stimulating cells during 5 hours with 30 ng/ml PMA and 500 ng/ml ionomycin (Sigma), in the presence of GolgiStop and brefeldin (BD Biosciences). Cells were surface-stained, fixed and permeabilized with BD Cytofix/Cytoperm and Perm/Wash (BD Biosciences) according to the manufacturer's instruction. Cells were incubated with PE-labeled antibody to IFNγ, IL-17F and CCL2; PECy7-labeled antibody to IL-10 (BD Biosciences), PerCPCy5.5-labeled anti- TNFα and CCL3. Detection of Ab FITC-labeled anti-CD107a (BioLegend) [34] were performed as previously described. Oxidation-sensitive dye H_2_DCFDA (Molecular Probes, Invitrogen), was used to measure ROS production [35]. Cells were acquired on FACSCanto II (BD Bioscience).

### Confocal microscopy

IHL were cultured in 24 wells on glass coverslips with ConA for 48 h, fixed in 4% paraformaldehyde, blocked with PBS-BSA 1% and labeled with FITC-anti mouse-CD3 and Alexa Fluor 647-anti- nitro tyrosine (NT), FITC-anti F4/80, rabbit anti- mouse-p47^phox^ and then incubated with Alexa Fluor 555- anti-rabbit. DNA was stained with DNA-binding fluorochrome Hoechst 33258 (2 ug/mL). Slides were observed with a FV1000 (Olympus) confocal microscope.

### q-PCR assays

DNA was isolated from 50 mg of hepatic tissue from I+LFD and I+MFD animals at 24 weeks of treatment using an *AccuPrep* Genomic DNA Extraction Kit (BIONNER), according to the manufacturer's protocol. Samples were stored at-80°C. The quantity and purity of the DNA were determined with the spectrophotometer Synergy HT Multi-Mode Microplate Reader (Biotek), and only samples with high purity were used in the experiments. 200 ng DNA were amplified by q-PCR using Power SYBR Green PCR Master Mix 2x (Applied Biosystems, Life Technologies) and specific primers for a *T*. *cruzi* satellite sequence. As an endogenous control, eEF2 primers were used [36]. Each primer pair was tested to reach the appropriate conditions of amplification. The primer sequence used were: Tcz1 (S: 5´-CGA GCT CTT GCC CAC ACG GGT GCT-3´ and Tcz2 (AS: 5´-CCT CCA AGC AGC GGA TAG TTC AGG-3´) [37]; and eEF2 (S: 5´-AAG CTG ATC GAG AAG CTG GA-3´ and AS: 5´-CCC CTC GTA TAG CAG CTC AC-3´).

Briefly, we amplified a 195pb TCZ satellite sequence using a initial denaturation step for 95°C for 10 min following of 40 PCR cycles with a denaturation temperature of 95°C for 15 s, an annealing and extension temperature of 60°C for 1 min, and a melting curve on a 7500 Real Time PCR System (Applied Biosystems). The internal standard eEF2 (186pb) was amplified with the same thermoprofile.

All DNA extractions and amplification reactions were performed with the appropriate negative controls to detect contamination at any stage of the procedure and with positive controls that gave reproducible results during all of the experiments and each sample was run by triplicate.

To determine levels of gene expression TCZ we used the comparative Ct method, known as 2^-ΔΔCT^ method [38].

### Statistical analysis

To compare different experimental conditions, analysis of variance (two-way or one-way ANOVA) along with Bonferroni's post test were performed using GraphPad software. A two-tailed Student' t-test was used for comparison between control and experimental samples. A p-value <0.05 was considered significant.

## Results

### *T*. *cruzi* infection reduces body weight, improves insulin resistance and increases the plasma triglyceride and cholesterol levels

**Figure 1:**
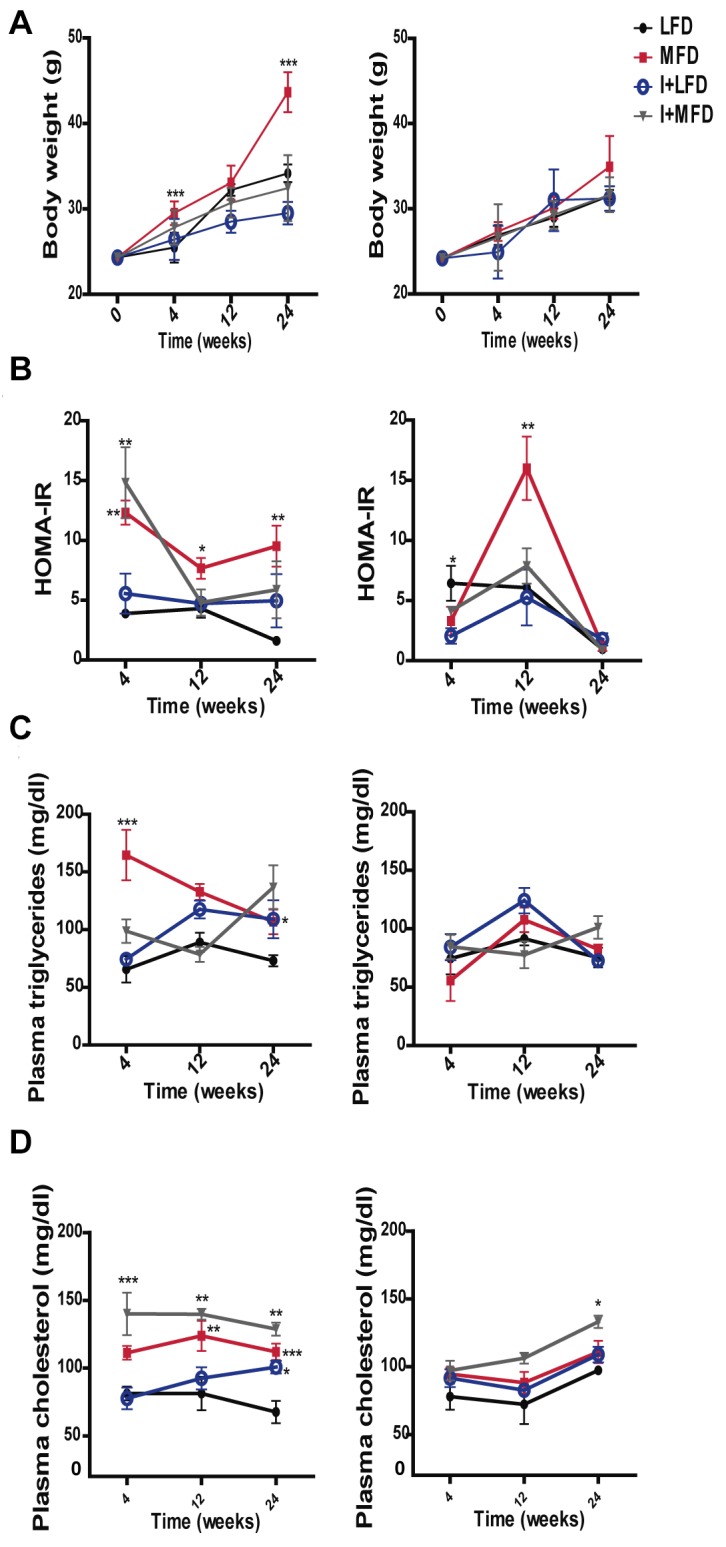
Metabolic abnormalities associated with diet-induced overweight and infection with *T*. *cruzi*. Metabolic parameters were measured after a 10-h fasting in mice on either MFD, LFD, I+MFD or I+LFD groups of WT and TLR4-/- mice at 4, 12 or 24 weeks, including: (**A)** Body weight ***p < 0.001 MFD vs. LFD at 4 and 24 weeks in WT; LFD vs. I+LFD **p < 0.01 at 12 and 24 weeks WT and ***p < 0.001 MFD vs. I+MFD at 24 weeks in WT. (**B)** Changes in plasma HOMA-IR in WT mice: **p < 0.01 MFD vs. LFD at 4 weeks, **p < 0.01 I+LFD vs. I+MFD at 4 weeks and MFD vs. LFD at 24 weeks, *p < 0.05 MFD vs. LFD at 12 weeks; Changes in plasma HOMA-IR in TLR4-/- mice: *p < 0.05 LFD vs. I+LFD at 4 weeks, ***p < 0.001 MFD vs. LFD, I+LFD and I+MFD at 12 weeks. (**C)** Plasma triglycerides concentrations in WT mice: ***p < 0.001 MFD vs. LFD at 4 weeks, **p < 0.01 MFD vs. I+MFD at 4 weeks. **(D)** Plasma cholesterol concentrations in WT mice: ***p < 0.001 I+LFD vs. I+MFD at 4 weeks; **p < 0.01 MFD vs. LFD and I+LFD vs. I+MFD at 12 weeks; ***p < 0.001 MFD vs. LFD at 24 weeks, *p < 0.05 LFD vs. I+LFD at 24 weeks and **p < 0.01 I+LFD vs. I+MFD at 24 weeks. The analysis in TLR4-/- groups: *p < 0.05 I+LFD vs. I+MFD and MFD vs. I+MFD at 24 weeks. n > 10 for each mice group were analyzed at all times studied. Data are shown as mean ± SEM of a representative assay from three independent experiments. A p-value <0.05 was considered significant using Two-way ANOVA test.

First, we analyzed biochemical and metabolic parameters of the experimental groups (LFD, MFD, I+LFD and I+MFD) in C57BL/6J WT and TLR4-/- mice. The WT group fed MFD had significantly higher body weight (43.5 ± 2.3 g) than LFD group (34± 1.0 g). Conversely, this increase on body weight was not observed in MFD TLR4-/- mice (33.7 ± 2.9 g). Notably, the weight due to MFD in WT mice was significantly reduced by the parasite infection (I+MFD) (32.4±3.7 g) (Fig. 1A). Next, we focused on the study of plasma glucose and insulin levels in all mice groups until 24 weeks of treatment. The (HOMA-IR) index, as an assessment of insulin resistance, is shown in Fig. 1B. This parameter was significantly increased in the MFD WT group (approximately 4 times higher than the LFD group) and remained elevated until 24 weeks. In the I+MFD WT group, this index increased early (4 weeks) but at the end of the study (24 weeks), was reduced compared to the MFD WT group. Noteworthy, only the MFD TLR4-/- group showed an increase of HOMA-IR at 12 weeks, although the values were similar for all TLR4-/- groups at the end of the study. The plasma triglyceride concentration in the MFD WT group was significantly higher than the LFD WT and I+MFD WT groups at 4 weeks. At 24 weeks, the triglyceride level was increased in all groups vs. the LFD WT. In contrast, there were no significant changes in triglyceride levels throughout the study in TLR4-/- groups (Fig. 1C). The mean values of plasma cholesterol at 4, 12 and 24 weeks from each group are presented in Fig. 1D. The highest cholesterol concentration was found in the I+MFD WT group at all studied time point. Later, at 24 weeks, cholesterol level was increased in all groups vs. the LFD WT group. In contrast, only I+MFD TLR4-/- group showed elevated cholesterol levels at 24 weeks compared to MFD TLR4-/- (Fig. 1D). To further analyze the lipoprotein distribution in plasma, the latter was submitted to agarose gel electrophoresis. In TLR4-/- mice, the lipoprotein pattern did not change among the groups, but an augmentation of LDL was noted in MFD WT mice at 24 weeks (S1 Fig.).

The MFD and LFD groups did not exhibit any clinical signs of illness during the entire experimental period, although the MFD group exhibited oily hair at 4 and 12 weeks. The I+LFD group displayed signs of illness such as squinting eyes, ruffled fur, reduced motility and appetite during the period of high parasitemia. In the chronic phase the clinical signs were more diffuse except for the loss of weight. In the I+MFD group these symptoms were more pronounced and appeared earlier, from day 20 dpi until the end of the experimental period.

### *T*. *cruzi* infection reduces the steatosis but enhances the hepatic inflammation

**Figure 2:**
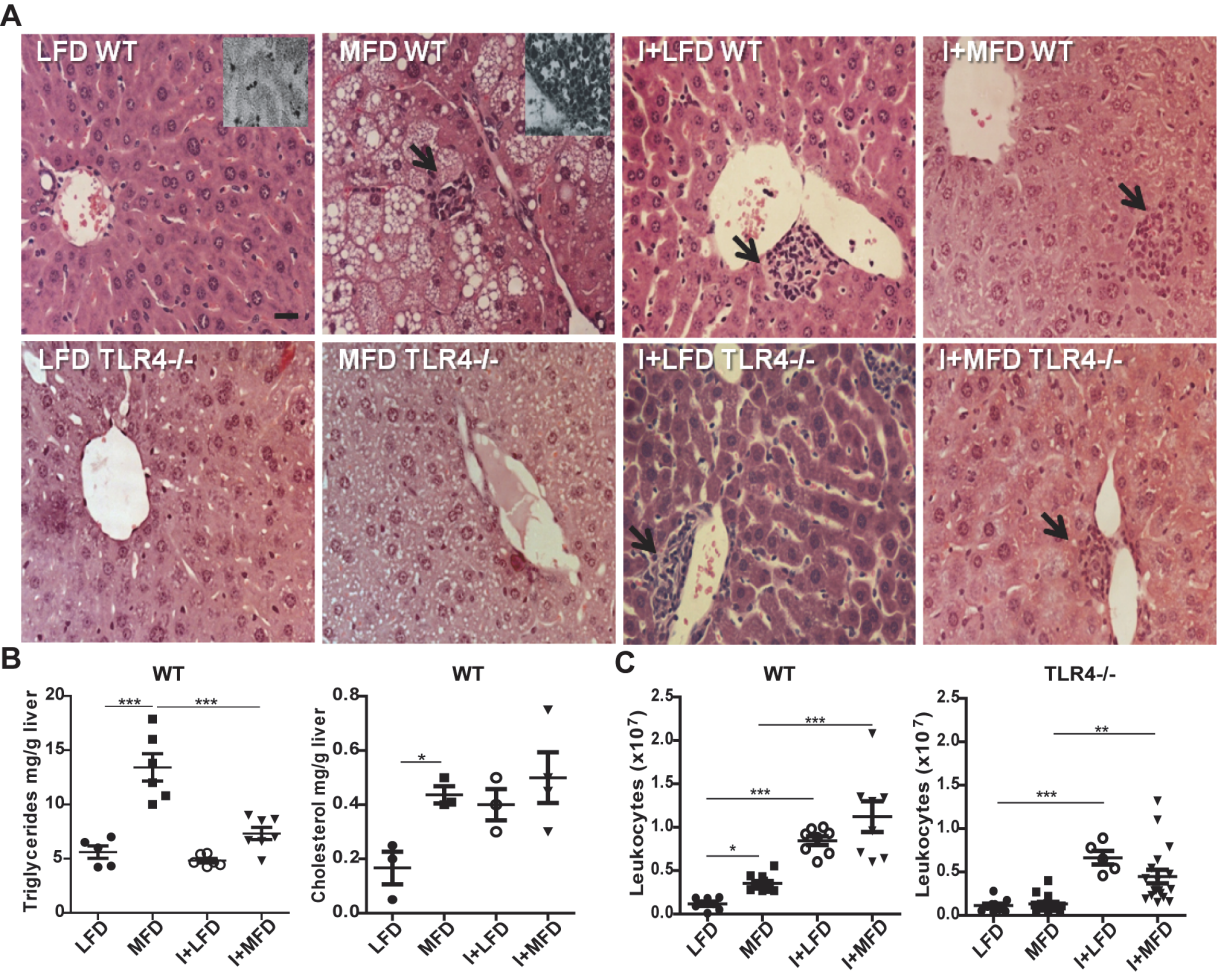
Hepatic inflammation and steatosis in WT and TLR4-/- mice with or without *T*. *cruzi* infection. **(A)** Liver tissue from all groups was subjected to hematoxylin and eosin (H&E at 400x) and Sudan Black IV staining (A, Insert) The arrows represent inflammatory foci. Scale bar = 20 μm. (**B)** Hepatic triglyceride and cholesterol contents were determined and expressed as mg/g of liver tissue of WT groups. (**C)** The numbers of IHLs were similar in mice of both genotypes with infection but the degree of steatohepatitis was significantly higher in MFD vs. LFD WT mice (*p < 0.05). All results are shown at 24 weeks of treatment and were representative of at least three independent experiments. Data are shown as mean ± SEM of more than 4 mice per group.

In the liver, the intake of MFD for 24 weeks produced lipid micro/macro vesicles and inflammatory foci, compatible with steatohepatitis only in WT mice. These morphological changes were not seen neither in the LFD WT fed mice nor in TLR4-/- groups. Notably, in the I+MFD WT or TLR4-/- group, we did not observe ectopic fat depots (Fig. 2A). These results were associated with high levels of hepatic triglyceride and cholesterol in the MFD WT group. However, the infection enhanced cholesterol accumulation in the liver (Fig. 2B). We did not found significant changes in hepatic triglyceride and cholesterol values between the different TLR4-/- groups (S2 Fig.). In addition, IHLs were found in both WT or TLR4-/- infected groups. This observation was further confirmed by hepatic leukocyte count (Fig. 2C).

### Parasite infection enhances the M1 inflammatory macrophage profile and CD36 expression

**Figure 3:**
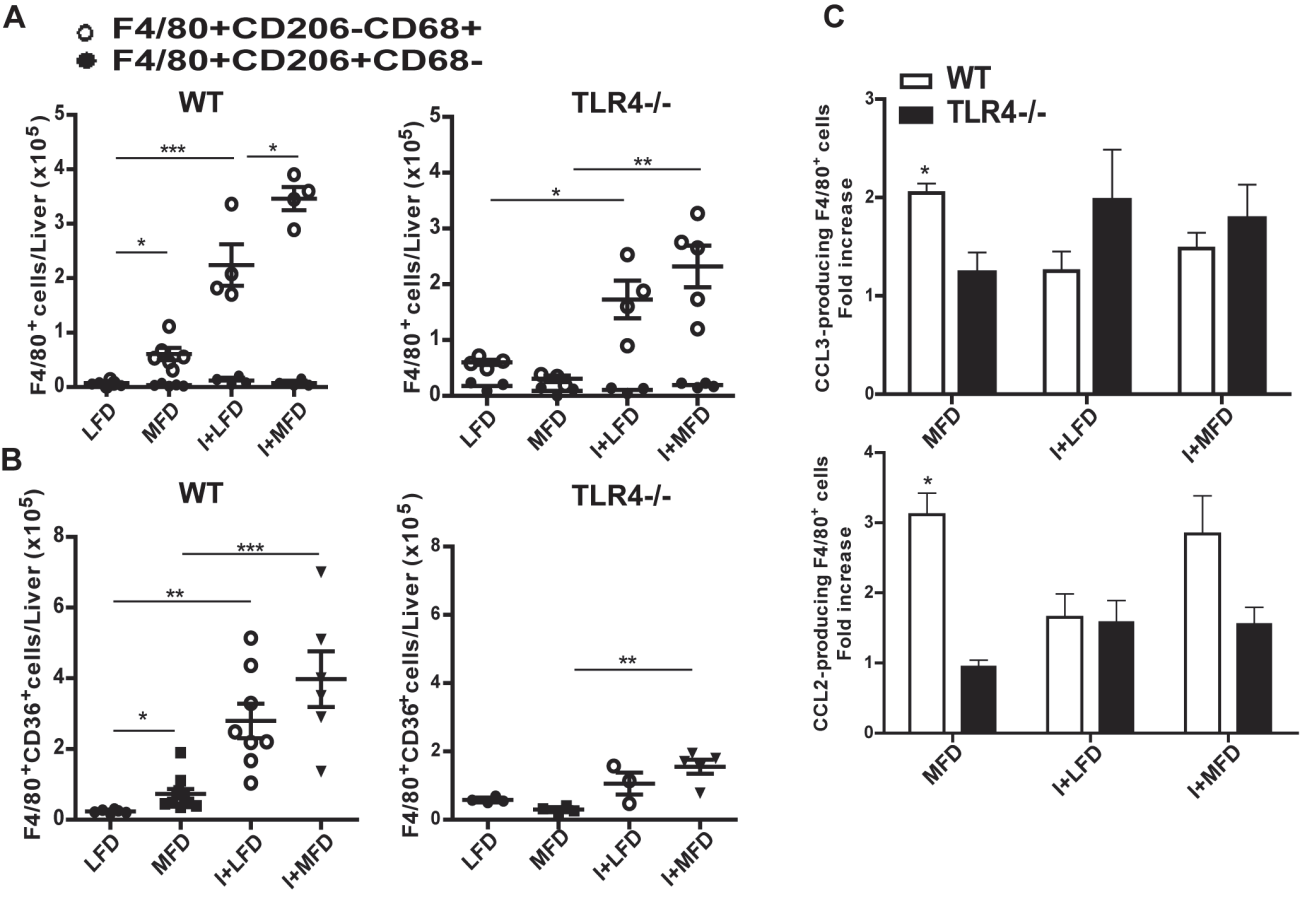
Expression of M1/M2 markers and CD36 expression from WT and TLR4-/- mouse liver macrophages. IHLs from all groups of mice were purified at 24 weeks and then stained with anti-F4/80, anti-CD206 anti-CD68 Abs. **(A)** The absolute number of F4/80^+^ cells of phenotype M1 (CD206^-^CD68^+^) and M2 (CD206^+^CD68^-^) are indicated. **(B)** The CD36 expression, after gating on F4/80 is showed. **(C)** Fold increase of percentages of CCL2- and CCL3-producing F4/80^+^ cells in liver of WT and TLR4-/- respect to LFD group. Cells were gated on F4/80 vs. side scatter dot plots. These assays were performed by flow cytometry and shown as mean ± SEM of six mice from one experiment representative of four performed.

It is widely known that F4/80^+^ cells play a critical role in liver inflammation. We found an increased F4/80^+^ cell number in MFD vs. LFD WT group at 24 weeks, whereas, the number of hepatic macrophage did not change in the MFD TLR4-/- group. However, the parasite infection increased the F4/80^+^ cell number in both WT and TLR4-/- groups. Moreover, increased absolute cell number and percentage of F4/80^+^CD206^-^CD68^+^ (Classical M1 macrophage polarization) were observed in experimental WT groups and infected TLR4-/-. The absolute number of F4/80^+^CD206^+^CD68^-^ cells (Alternative M2 macrophage polarization) was similar among the different WT groups although the percentages diminished in all groups vs. LFD WT (Fig. 3A and S3 Fig.). Conversely, the MFD TLR4-/- mice did not show any differences in the number of M1 cells vs. LFD TLR4-/- mice (Fig. 3A). In order to better characterize the macrophage population, we also included CD11c marker to discriminate F4/80^+^CD206^-^CD11c^+^ (M1) and F4/80^+^CD206^+^CD11c^-^ (M2) macrophages with similar results to those described above (S4 Fig.). In addition, we observed a high frequency of F4/80^+^CD11c^+^TNFα^+^ cells in all WT groups vs. the LFD WT (S4 Fig.).

CD36 has been highly implicated in the metabolic inflammatory disease [28,29], hence, we analyzed the expression pattern of this scavenger receptor on F4/80^+^ cells. We observed an increased CD36 expression in all WT groups with the highest values in I+MFD and I+LFD groups. We did not observe any changes when compared MFD vs. LFD TLR4-/- mice however, infected TLR4-/- groups showed augmented CD36 expression on F4/80^+^ cells (Fig. 3B). Additionally, we detected a significant fold increase of CCL3/MIP-1α and CCL2/MCP-1 producing F4/80^+^ cells only in the WT strain fed with MFD (Fig. 3C).

### *T*. *cruzi* infection exacerbates the local T cell response induced by MFD

**Figure 4:**
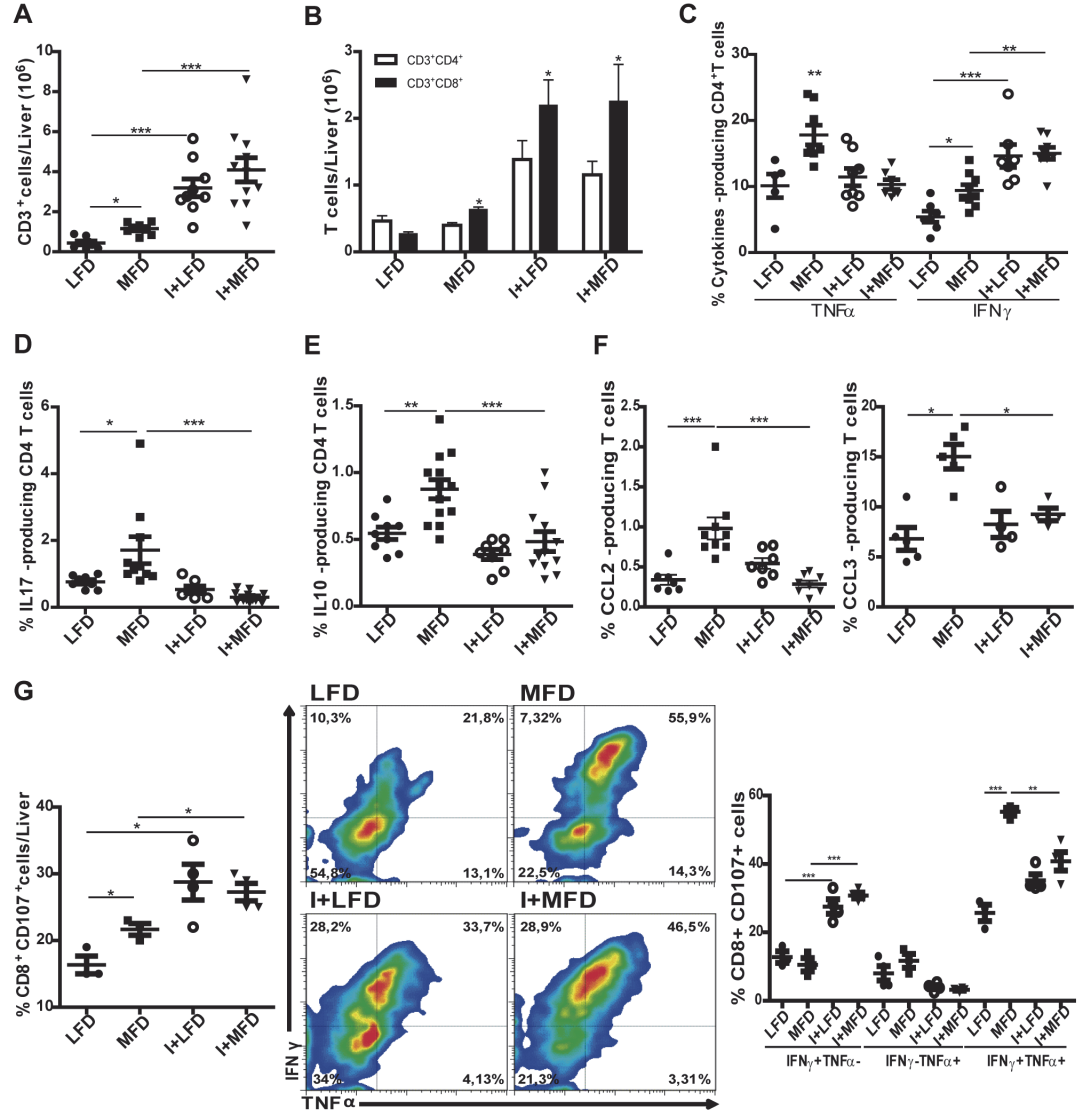
The local T cell response is induced by MFD and exacerbated by *T*. *cruzi* infection. IHLs from different groups of mice obtained at 24 weeks were stained with anti-CD3, anti-CD4, anti-CD8, anti-TNFα, anti-IFNγ, anti-IL17, anti-IL10, anti-CCL2, anti-CCL3 and anti-CD107 Abs. **(A)** The absolute numbers of CD3^+^ and **(B)** the absolute numbers of CD3^+^CD4^+^ and CD3^+^CD8^+^ cells in liver are indicated. **(C)** Percentage of TNFα and IFNγ producing CD4^+^ T cells in liver of WT are shown by intracellular staining. Percentage of **(D)** IL17 and **(E)** IL10 producing CD4^+^ T cells in liver of WT are shown by intracellular staining. **(F)** Percentages of CCL2 and CCL3 producing CD3+ T cells in liver of WT are shown by intracellular staining. **(G)** Percentages of CD107a^+^CD8^+^ T cells. IHLs from all groups were cultured in the presence of PMA plus ionomycin and monensin for 5 h and stained with corresponding antibodies. Data are shown as mean ± SEM of more than 4 mice per group from one experiment representative of three performed.

Next, we analyzed the total number of CD3^+^ cells and CD4^+^/CD8^+^ subpopulations in the liver of WT mice at 24 weeks. We observed a high number of T cells in all experimental groups with the highest values in I+MFD group (Fig. 4A). The MFD WT group increased CD3^+^CD8^+^ cell number, and more prominently the infected groups (Fig. 4B). To better characterize the subtype of T helper cells we analyzed the production of IFNγ/TNFα and IL-17 (Th1 and Th17 respectively) or IL10 (Th2 phenotype). We observed a high number of IFNγ-producing CD4^+^ T cells in all experimental groups vs. LFD WT, as it is shows in Fig. 4C. Surprisingly, our results revealed a strong increment of TNFα (Fig. 4C) and IL17- producing T cells only in the MFD WT group (Fig. 4D). In addition, CD4^+^ IL10^+^ T cells were significantly increased in the MFD WT group (Fig. 4E). Regarding CCL2 and CCL3, two important chemokines for recruitment and activation of monocytes/macrophages, we found a significant increase of CCL2- and CCL3-producing T cells only in MFD WT group (Fig. 4F). Strikingly, TLR4-/- mice did not reveal any difference between the MFD and LFD groups (S5 Fig.). Finally, we observed intracellular staining for IFNγ and TNFα in CD8^+^CD107^+^ T cells in all WT groups as shown in Fig. 4G. Remarkably, the MFD group showed an elevated number of TNFα^+^ and IFNγ^+^ double positive cells while infected groups showed an increased number of IFNγ^+^ single positive and TNFα^+^ and IFNγ^+^ double positive cells compared to the LFD WT group.

### The recruitment of B lymphocytes, NK and dendritic cells in liver is induced by MFD and is exacerbated by parasite infection

**Figure 5:**
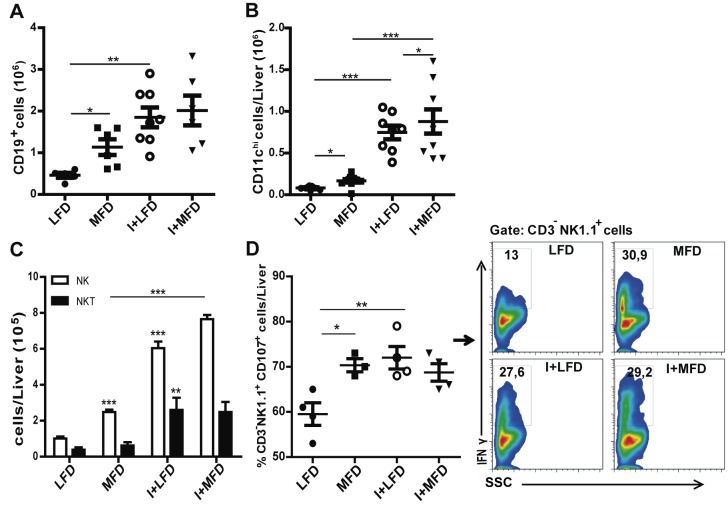
The recruitment of B lymphocytes, dendritic cells and NK cells in liver is induced by MFD and increased by parasite infection. IHLs from different groups of mice were stained with anti-CD19, anti-CD11c, anti-CD3, anti-NK1.1, anti-CD107 and anti-IFNγ. **(A)** The absolute numbers of CD19^+^, **(B)** the absolute number of CD11c^hi^ in liver and **(C)** the absolute number of NK and NKT cells are indicated. **(D)** Percentage of CD3^-^ NK1.1^+^ CD107a^+^ cells. IHLs from all groups were cultured in the presence of PMA plus ionomycin and monensin for 5 h and stained with corresponding antibodies. Data are shown as mean ± SEM of more than 4 mice per group from one experiment representative of three performed.

We also found a high number of CD19^+^, CD11c^+ high^, and NK1.1^+^CD3^-^ (NK) cells in liver of MFD WT mice (Fig. 5A-C). The increment of these cell populations was more pronounced in infected mice (I+LFD or I+MFD WT groups). On the other hand, NK1.1^+^CD3^+^ (NKT) cells were increased in infected groups but not in MFD WT mice. In addition, IFNγ-producing NK cells from all experimental WT groups showed a CD107^+^ activated phenotype compared with the LFD WT group (Fig. 5D). Notably, we did not observe any difference in MFD TLR4-/- mice, demonstrating the key role of TLR4 in our steatohepatitis model. However, the infected TLR4-/- groups showed an increased number of these cell populations (S6 Fig.).

### The outburst of systemic inflammatory cytokines is highly dependent on TLR4 signaling

**Figure 6:**
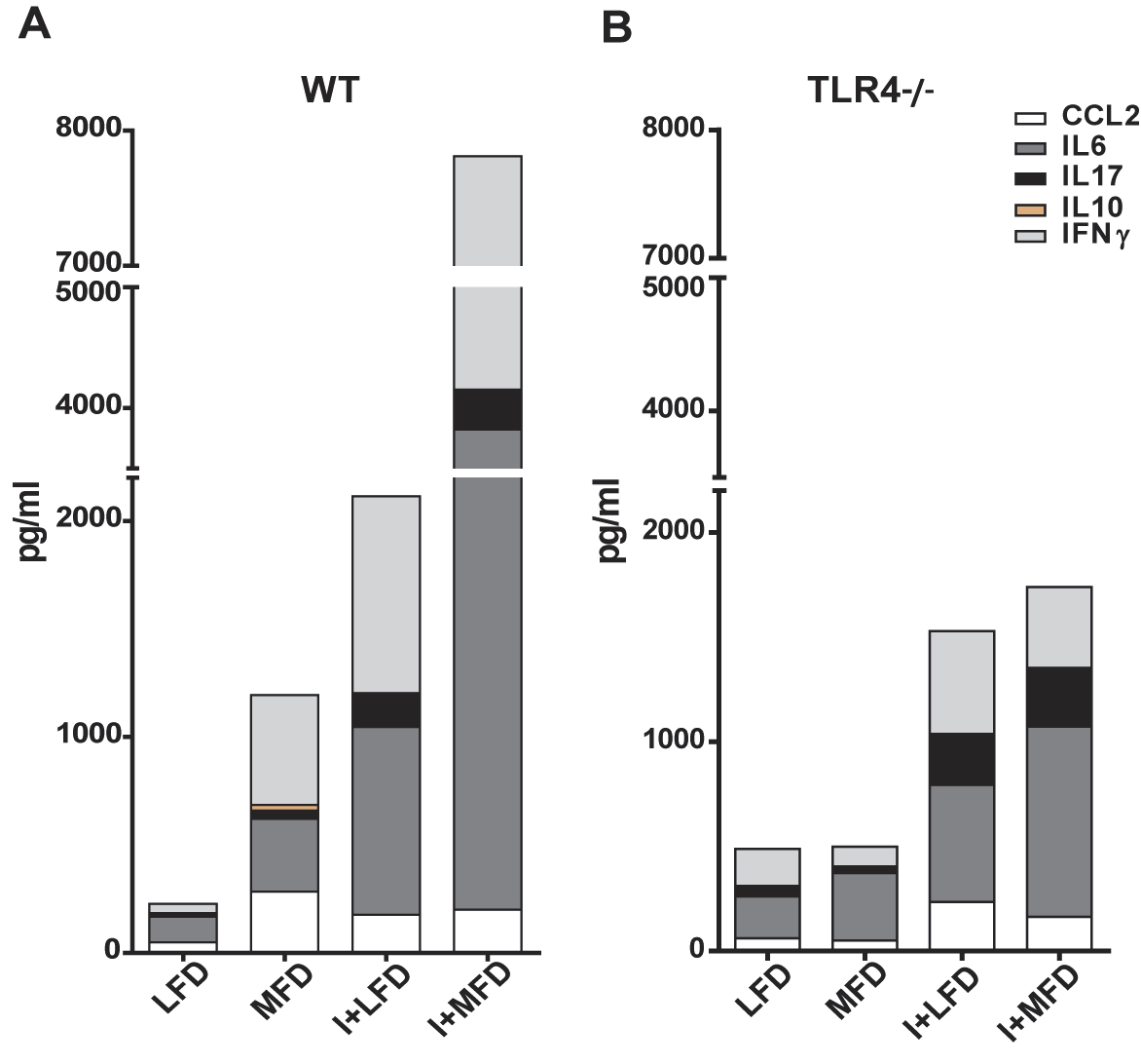
The outburst of systemic inflammatory cytokines is highly dependent on TLR4 signaling and increased by parasite infection. The cytokine production was quantified by ELISA in plasma collected from all groups of mice at 24 weeks of treatment with LFD, MFD, I+LFD or I+MFD. **(A)** WT CCL2: **p < 0.01 MFD vs. LFD and *p < 0.05 I+LFD vs. LFD; IL6: *p < 0.05 MFD vs. LFD, **p < 0.01 I+LFD vs. LFD, ***p < 0.001 MFD vs. I+MFD and **p < 0.01 I+LFD vs. I+MFD; IFNγ: *p < 0.05 MFD vs. LFD, **p < 0.01 I+LFD vs. LFD, **p < 0.01 MFD vs. I+MFD and *p < 0.05 I+LFD vs. I+MFD; IL17: **p < 0.01 MFD vs. LFD, **p < 0.01 I+LFD vs. LFD, ***p < 0.001 MFD vs. I+MFD and *p < 0.05 I+LFD vs. I+MFD. **(B)** TLR4-/- CCL2: *p < 0.05 I+LFD vs. LFD; IFNγ: *p < 0.05 MFD vs. I+MFD; IL17: **p < 0.01 I+LFD vs. LFD and ***p < 0.001 MFD vs. I+MFD. All data are shown as mean ± SEM of four to eight mice per group from one experiment representative of two performed. A p-value <0.05 was considered significant using Two-way ANOVA test.

Lipid accumulations, their subsequent lipotoxicity and parasite infection trigger intracellular signaling pathways, which lead to the production of pro-inflammatory cytokines and chemokines and are responsible for cellular recruitment. In our model, we detected high levels of plasmatic IL6, IL17, IFNγ cytokines and the CCL2 chemokine in all experimental WT groups vs. LFD at 24 weeks; the highest levels of IL6 and IFNγ were observed in I+MFD vs. MFD WT (about 100 fold increase). In addition, the IL10 anti-inflammatory cytokine was only detected in the plasma of the MFD WT (Fig. 6A). The concentrations of inflammatory cytokines were very low in plasma of infected TLR4-/- mice in comparison with their respective control WT groups, as shown in Fig. 6B. Likewise, the MFD TLR4-/- group did not show differences compared to the LFD TLR4-/- group.

### Parasite infection exacerbates reactive oxygen species and peroxinitrites production by liver inflammatory leukocytes in MFD group

**Figure 7:**
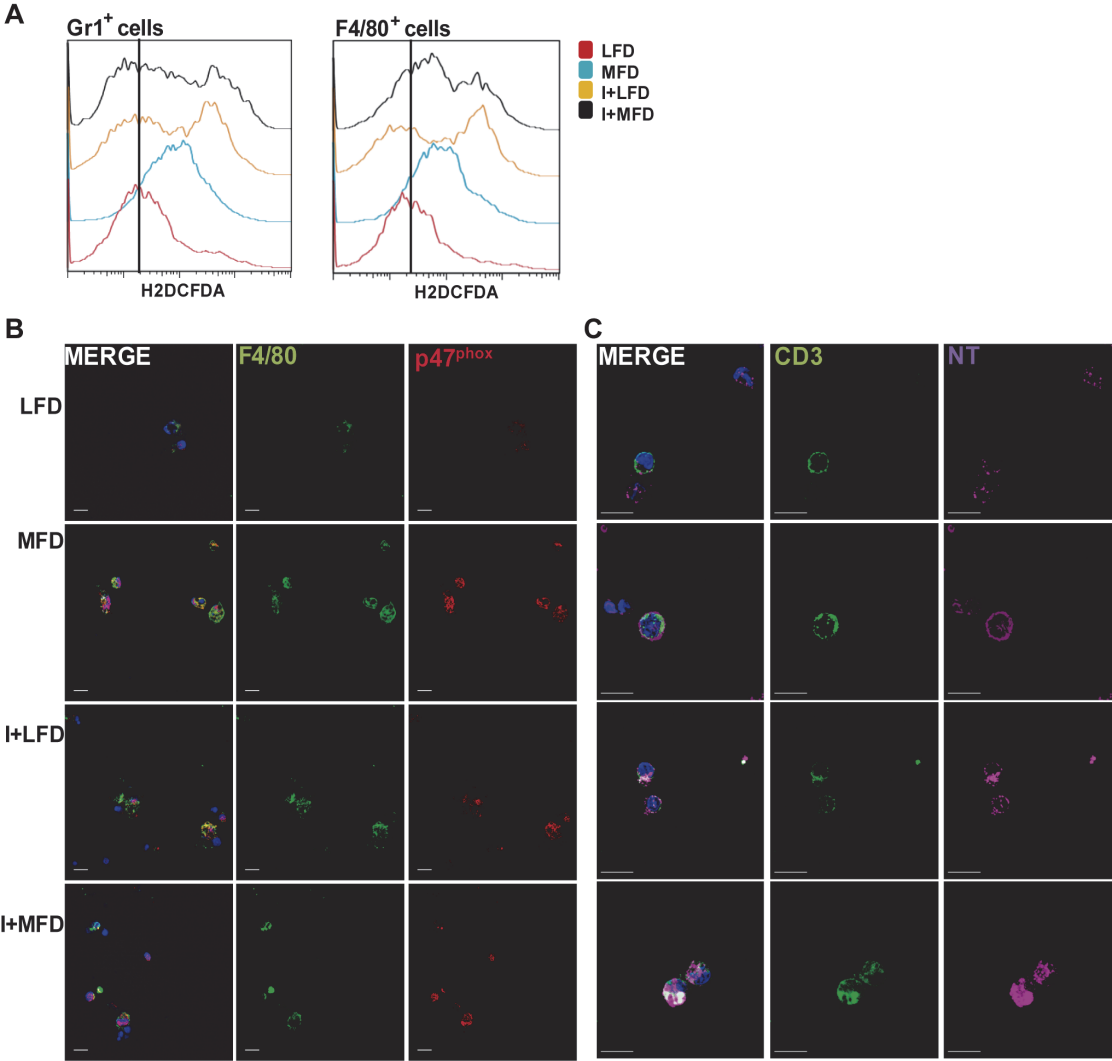
Reactive oxygen species and peroxinitrites are produced by liver inflammatory leukocytes induced by MFD and exacerbated by parasite infection. **(A)** IHLs from all groups WT were stimulated with PMA (30 mg/mL) and incubated with H2DCDFA (10uM) for 30 minutes at 37°C and Intrahepatic cells were then stained with anti-Gr-1, anti-F4/80. The percentage of ROS in Gr-1^+^ and F4/80^+^ cells was measured by flow cytometry and the results are shown as mean ± SEM of six mice from one experiment representative of two performed. Statistical significance determined by one-way ANOVA test. **(B)** Expression of p47^phox^ in F4/80^+^ cells. IHLs from different groups of mice were stimulated with Con A 48h and then stained with anti-F4/80 FITC, anti-p47^phox^/Alexa Fluor 555 Abs and then visualized using confocal microscopy. **(C)** Expression of tyrosine nitration in CD3^+^ T cells. IHLs from different groups of mice were stimulated with Con A 48h and then stained with anti-CD3 FITC and anti-TN/Alexa Fluor 647 Abs, and visualized using a FV300 Olympus confocal microscope. Scale bar = 10 μm.

Taking into account the key role of macrophages and T lymphocytes during steatohepatitis, we first evaluated the ROS production by liver- infiltrating F4/80^+^ and Gr1^+^ cells by comparing their H_2_DCFDA fluorescent signals. Increased amount of ROS were produced in all experimental groups compared with the LFD WT as shown by their respective histograms (Fig. 7A). Moreover, an increased expression of cytoplasmic p47^phox^, a subunit of NADPH oxidase complex, was observed (Fig. 7B). We also demonstrated a high expression of tyrosine nitration on T-cell surface (Fig. 7C). Furthermore, protein tyrosine nitration occurred in hepatic tissue of I+MFD WT group was noted suggesting that peroxynitrite radicals or derivatives may contribute to hepatic injury. These results were associated with a high expression of FasL on hepatic CD3^+^ cells from all experimental groups compared with LFD group (S7 Fig.).

### Medium-fat diet leads to increased parasite load in the liver

**Figure 8:**
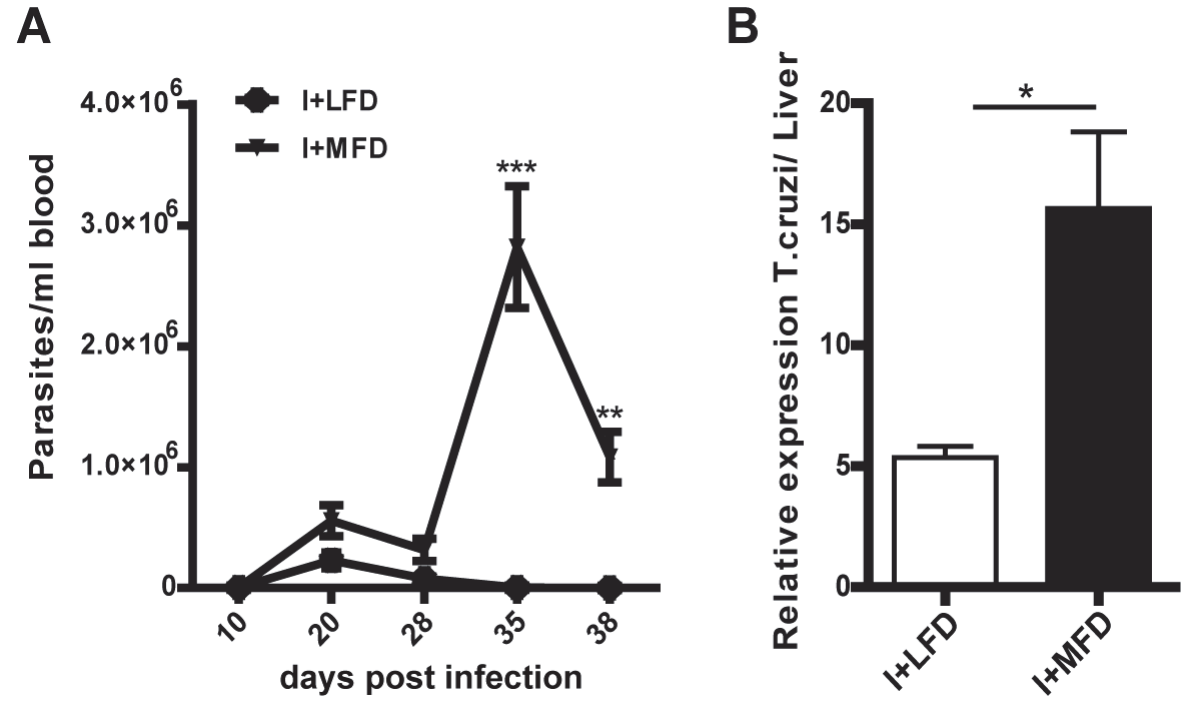
Medium-fat diet leads to increased parasitemia and parasite load in the liver. **(A)** Parasitemia was determined at different time post *T*. *cruzi* infection in I+LFD and I+MFD WT mice, n = 10 per group. **(B)** Quantitative assessment of the parasite load by q-PCR in the liver of infected groups. Parasite quantification in DNA samples of I+LFD and I+MFD groups was performed amplifying a TCZ *T*. *cruzi* satellite sequence at 24 weeks post treatment. The relative load of parasites/liver was normalized against the eEF2 housekeeping gene control. Two positive samples, two negative samples and non-template DNA were included in every q-PCR. All data are shown as mean ± SEM of four mice per group from one experiment representative of two performed. A p<0.05 was considered significant using two-tailed T test.

Given the strong inflammatory response at local and systemic level found in I+MFD group we investigated whether the parasitemia and/or hepatic parasite load were influenced by the type of diet. The parasitemia was detected until 38 days post-infection, and was higher in I+MFD than in I+LFD group (Fig. 8A). Given that the parasitemia was undetectable at the chronic phase, we determined by q-PCR *T*. *cruzi* DNA in livers of infected groups. The parasite load was higher in I+MFD than I+LFD animals (Fig. 8B). These observations revealed that the diet is a key factor in the liver parasite burden and hepatic injury.

## Discussion

It is well known that IR commonly coexists with obesity, hypertension, hyperglycemia and dyslipidemia involving elevated triglycerides, small dense low-density lipoprotein particles, and decreased HDL cholesterol levels. However, causal links between IR, obesity, and dietary factors are complex and controversial. High plasma insulin and glucose levels due to IR are the major components of the metabolic syndrome [39]. Our results demonstrate that a medium fat diet alone is able to induce weight gain and IR. The highest HOMA-IR values were detected in the MFD group at 4 weeks and remained elevated until 24 weeks. As a result of IR, increased hepatic and plasma triglycerides and plasma LDL levels were also detected in the MFD group (Supl.1). In this sense, it was reported that high TNFα concentrations increased plasma triglycerides levels by stimulating hepatic lipid secretion [40]. Besides, this cytokine is characterized by possessing elevated lypolitic activity in adipocytes and by making body tissues more resistant to the effects of insulin in different disease states [41,42]. Interestingly, this parasite may persist in adipose tissue of humans and mice and become a reservoir of infection [43]. Our results clearly demonstrate that parasite infection induced marked metabolic changes such as a reduction of HOMA-IR and fat accumulation in hepatic tissue with marked decrease in hepatic triglyceride content at 24 weeks. Recently, Tanowitz's group described that *T*. *cruzi* Brazil strain infection induced glucose homeostasis changes, pancreatic inflammation and parasitism within pancreatic β cells [44] but the effect of the Tulahuen strain in the pancreas is yet unknown. Alternatively, the parasite could require host lipoproteins in order to multiply inside of cells [45]. Moreover, parasite-derived lipid metabolizing enzymes could play a variety of roles during invasion, survival, establishment and exacerbation of the disease [46]. Noteworthy, we observed that the liver remained a prominent reservoir of parasites at the chronic stage, at 24 weeks post-infection. Several observations suggest that *T*. *cruzi* take advantage from host cell metabolism that favors fatty acid oxidation over glucose oxidation [45]. Moreover, proteomic evidence shows that *T*. *cruzi* upregulates the ability for fatty acid uptake and oxidization, suggesting a coupling of parasite growth to fatty acid metabolism in the host [47]. Interestingly, we also observed that the increase in body weight due to a MFD was suppressed by parasite infection (I+MFD) at 12 and 24 weeks concordantly with the results reported by other researchers [32]. Besides, the blood triglyceride levels were also diminished by the infection at 4 weeks compared with its respective control (MFD group). Strikingly, the infection or MFD or the combination of both variables (I+MFD) increased the plasmatic and hepatic cholesterol concentrations. In this sense, it was postulated that the *T*. *cruzi* infection induced changes in intracellular cholesterol homeostasis, since it was reported an accumulation of LDL and cholesterol in tissues during both acute and chronic infection [48]. It was also demonstrated that this parasite may use the LDL receptor for cell invasion [49]. As expected, the MFD TLR4-/- group showed minor metabolic changes compared to the MFD WT group without changes in body weight [26,50]. Notably, the infection was able to increase plasmatic cholesterol levels in MFD TLR4-/- mice at a later time (24 weeks).

On the other hand, our results were consistent in demonstrating that the combination of *T*. *cruzi* infection with MFD induced more liver inflammation in WT than in TLR4-/- mice. Low systemic cytokine levels and reduced number of intrahepatic leukocytes were observed in TLR4-/- groups. The pro-inflammatory cytokines may disrupt normal insulin action in fat and muscle tissue and could be a major factor in causing the IR. Furthermore, it is known that fatty liver may be more susceptible to injury because of leukocyte infiltration. Our results demonstrate a predominant M1 F4/80^+^ cell accumulation in the liver from the MFD group only in WT mice suggesting that inflammatory macrophage polarization is dependent on TLR4 signaling. Strikingly, parasite infection markedly enhanced this phenomenon in two mouse strains (WT and TLR4-/-). Thus, the combination of *T*. *cruzi* infection with fat diet (I+MFD) exacerbated M1 profile (F4/80^+^CD11c^+^TNFα^+^) in WT mice (S2 Fig.). The recruitment of these cells in NASH was consistent with the high levels of CCL2 and CCL3 chemokines in liver and blood, as detected by us and others [51,52]. Numerous evidence indicates that TNFα, CCL2 and CCL3 are also produced during the course of *T*. *cruzi* infection [53,54], which could explain the great recruitment of inflammatory cells into the hepatic tissue.

Interestingly, we detected an increase of CD36 scavenger receptor on hepatic F4/80^+^ cells of the MFD WT group and even more of infected groups. Since, members of the CD36 scavenger receptor family have been implicated as sensors of microbial products that mediate phagocytosis and inflammation; we speculated that *T*. *cruzi* PAMPs could directly interact with this receptor. The presence of a lipoprotein scavenger-like receptor in the parasite cannot be discarded and this mechanism could also explain the reduction of fat accumulation in the liver of I+MFD vs. MFD WT groups. Furthermore, our data unequivocally show that IHL such as B lymphocytes, DCs and IFNγ^+^-producing activated cytotoxic T cells and NK were increased during the progression of steatohepatitis in the MFD group and notably, the parasite infection had a remarkable synergistic effect in this process (24 weeks).

On the other hand, IL10-producing T lymphocytes, likely Th2 and/or T regulatory infiltrating liver, and plasma IL10, were only detected in the MFD WT group. The protective role of IL10 in IR may be involved in this step of steatohepatitis, balancing the effect of pro-inflammatory cytokines. Conversely, in the I+MFD group, this cytokine was not detected, indicating that the parasite infection produced a marked deregulation of this anti-inflammatory mechanism [55]. In turn, high circulating levels of pro-inflammatory cytokines were detected in MFD WT mice as well as in obese subjects [56] which may contribute to IR and to low-grade systemic inflammation. It is also known that pro-inflammatory cytokines play an important role in controlling the parasite replication [57]. As expected, TNFα, IL6 and IFNγ cytokines were enhanced by the infection, which have been associated with the progression of hepatic [18] and cardiac injury [58]. The inflammatory cytokine profiles were more attenuated in TLR4-/- groups, either in the physiological steatohepatitis model alone or in combination with *T*. *cruzi* infection. The IL6 role in NASH is controversial since it was postulated that is a key factor in the progression of this disease and the development of IR [59] and other researchers have assumed that IL6 may limit endotoxemia and obesity-associated IR [60]. We claimed that the strong IL6 production found in this model would appear limiting the development of IR. In addition, in a similar way that occurs in humans, we found an increased number of IL17 ^-^producing CD4 T cells in the MFD WT group, although other sources of IL17 like γδ T or NKT cells could be involved. Furthermore, IL-17 and FFAs synergize to produce IL6 by hepatocytes [61] and IL17/IL17RA signaling is essential for controlling *T*. *cruzi* infection [62]. However, IL-17 signaling accelerates the progression of NASH in mice [63]. Thus, this study demonstrates for the first time a synergistic role of IL-17 in steatohepatitis associated with this infection.

An intense cross-talk between hepatocytes, leukocytes and cytokines, added to oxidative stress and apoptosis, are key actors in the steatohepatitis pathogenesis [64]. In this scenario, our results demonstrated an increased ROS production by liver macrophages with tyrosine nitrated proteins and an enhanced FasL expression on T-cell surface, which may induce apoptosis of hepatocytes. Interestingly, the parasite infection exacerbated oxidative stress in our NASH model.

Taken together, these findings highlight the contribution of our laboratory in understanding how *T*. *cruzi* infection is able to diminish triglycerides accumulation in liver, while at the same time, exacerbates the inflammatory process in a physiological NASH model. New tools that integrate current knowledge of steatohepatitis with American Trypanosomiasis pathogenesis must be considered in the future.

## Supporting Information

S1 Fig Plasma lipoprotein distribution of WT and TLR4-/- mice induced by diet or/and *T*. *cruzi* infection. Lipoprotein distribution were assessed at 24 weeks by agarose gel electrophoresis, after a 10-h fasting in mice on either LFD, MFD, I+LFD or I+MFD groups WT and TLR4-/- mice.
(TIF)

S2 Fig Hepatic triglyceride and cholesterol contents were similar among different TLR4-/- groups. Hepatic triglyceride and cholesterol contents were determined and expressed as mg/g of liver tissue in TLR4-/- groups. The results are shown at 24 weeks of treatment and were representative of at least three independent experiments. Data are shown as mean ± SEM of more than 3 mice per group.
(TIF)

S3 Fig Shift of macrophage phenotype in experimental WT groups. Percentages of hepatic F4/80^+^ cells of phenotype M1 (CD206^-^CD68^+^) and M2 (CD206^+^CD68^-^) are indicated. Data are shown as mean ± SEM of more than 3 mice per group from one experiment representative of three performed.
(TIF)

S4 Fig Frequency of F4/80+ CD11c+ macrophages (M1) positively stained for TNFα in different WT groups. IHLs obtained at 24 weeks were stained with anti-F4/80, anti-CD11c, anti CD206 and anti-TNFα. **(A)** Percentage of F4/80^+^ CD206^-^ CD11c^+^ cells of different groups of WT mice at 24 weeks is indicated. **(B)** IHLs from all groups were cultured in the presence of PMA plus ionomycin and brefeldin for 5 h and stained with anti-F4/80, anti-CD11c and anti-TNFα. A representative graphic is shown. Data are shown as mean ± SEM of more than 4 mice per group from one experiment representative of three performed.
(TIF)

S5 Fig Changes in hepatic T cell phenotype of TLR4-/- mice groups. IHLs at 24 weeks were stained with anti-CD3, anti-CD4, anti-CD8, anti-TNFα and anti-IFNγ Abs. (**A)** The absolute numbers of CD3^+^ and **(B)** the absolute number of CD3^+^CD4^+^ and CD3^+^CD8^+^ cells in liver is indicated. **(C)** Percentage of TNFα and IFNγ producing CD4^+^ or CD8^+^ T cells in liver are shown by intracellular staining. IHLs from all groups were cultured in the presence of PMA plus ionomycin, monensin and brefeldin for 5 h and stained with corresponding antibodies. The results are expressed as mean ± SEM of more than 4 mice per group from one experiment representative of three performed.
(TIF)

S6 Fig The recruitment of B lymphocytes, dendritic cells and NK cells in liver TLR4-/- animals was not influenced by MFD but it was increased by parasite infection. IHLs from different groups of mice were stained with anti-CD19, anti-CD11c, anti-CD3, anti-NK1.1. **(A)** The absolute numbers of CD19^+^, **(B)** the absolute number of CD11c^hi^ in liver and **(C)** the absolute number of NK and NKT cells are indicated. Data are shown as mean ± SEM of more than 3 mice per group from one experiment representative of three performed.
(TIF)

S7 Fig Protein tyrosine nitration in hepatic tissue and expression of FasL on CD3^+^ cells in experimental WT mice. **(A)** 400x micrographs of hepatic tissue section allow panoramic evaluation of protein tirosine nitration after stained with Ab Alexa Fluor 488-anti- nitro tyrosine (NT). Photographs are representative of one out of five mice. **(B)** IHL were cultured with ConA for 48 h and labeled with FITC-anti mouse-CD3 and PE-anti FasL. DNA was stained with DNA-binding fluorochrome Hoechst 33258 (2 ug/mL). Slides were observed with a FV1000 (Olympus) confocal microscope. Scale bar: 10 μm.
(TIF)
