# Supplementary figures and images for: Trypanosoma cruzi Infection Is a Potent Risk Factor for Non-alcoholic Steatohepatitis Enhancing Local and Systemic Inflammation Associated with Strong Oxidative Stress and Metabolic Disorders
Source: PLoS Negl Trop Dis. 2015 Feb 10;9(2):e0003464. doi: 10.1371/journal.pntd.0003464 (PMC4323252; doi:10.1371/journal.pntd.0003464)

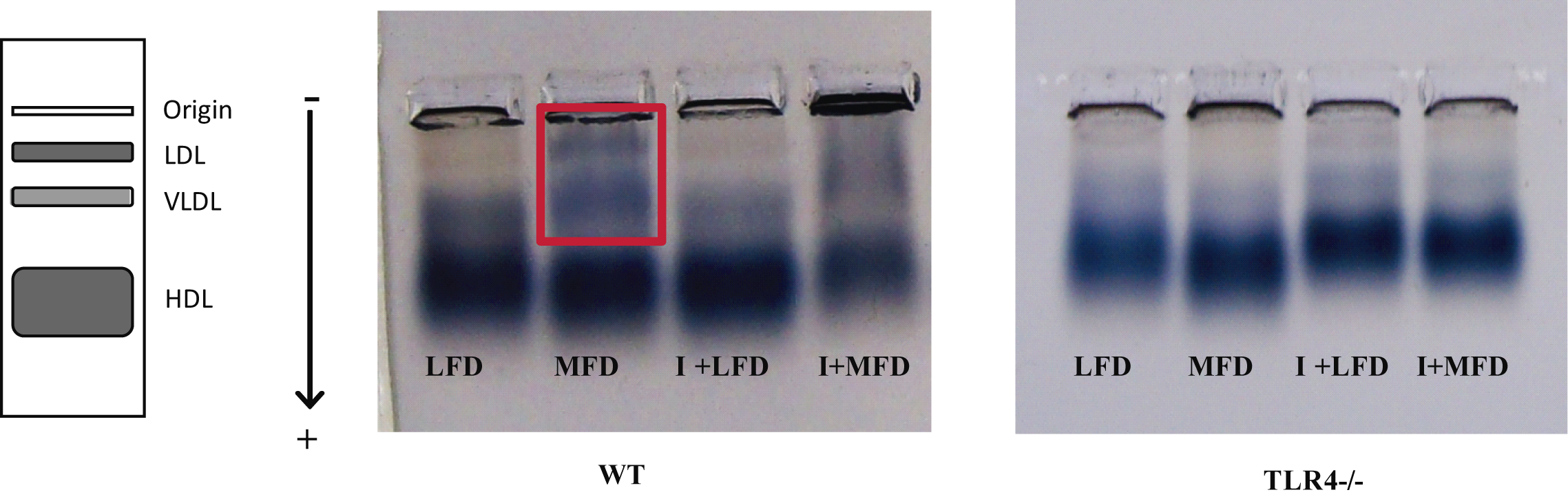

Supplement: S1 Fig — Lipoprotein distribution were assessed at 24 weeks by agarose gel electrophoresis, after a 10-h fasting in mice on either LFD, MFD, I+LFD or I+MFD groups WT and TLR4-/- mice. (TIF) [file pntd.0003464.s001.tif]

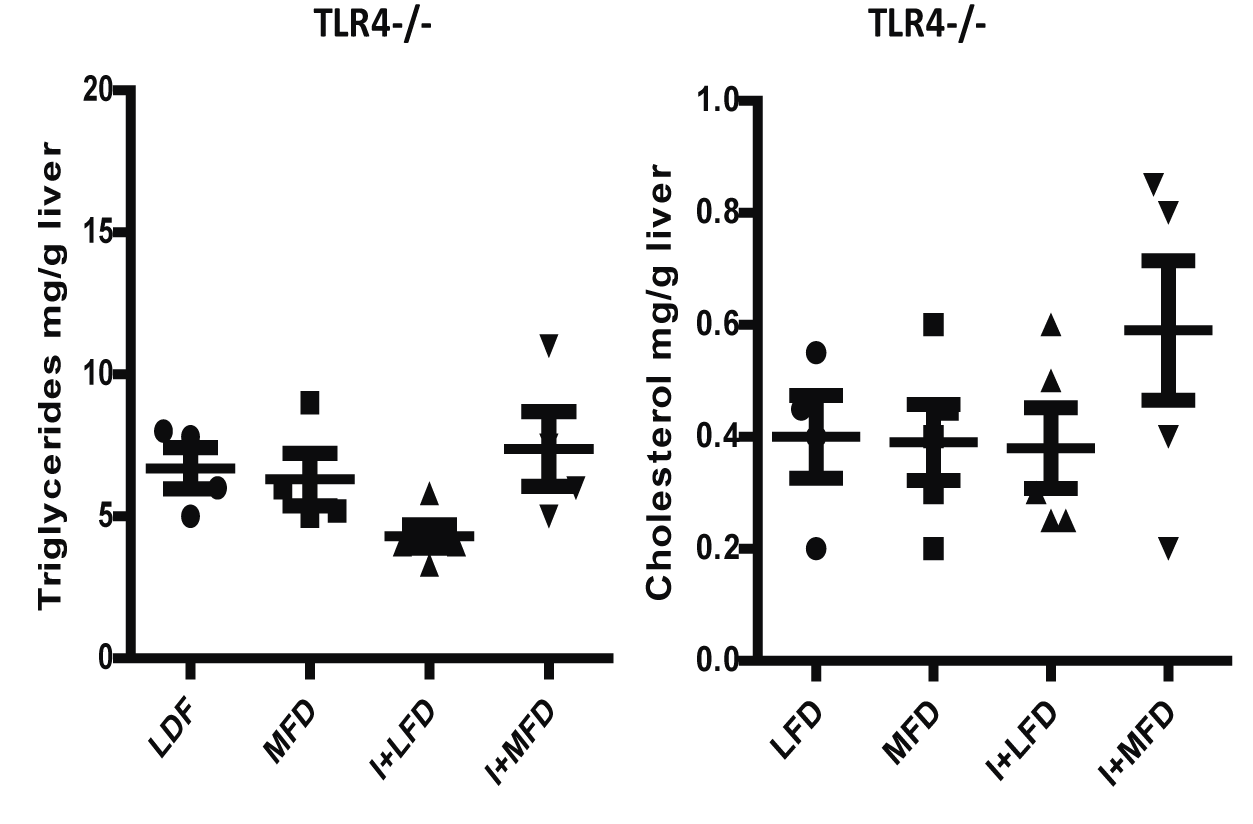

Supplement: S2 Fig — Hepatic triglyceride and cholesterol contents were determined and expressed as mg/g of liver tissue in TLR4-/- groups. The results are shown at 24 weeks of treatment and were representative of at least three independent experiments. Data are shown as mean ± SEM of more than 3 mice per group. (TIF) [file pntd.0003464.s002.tif]

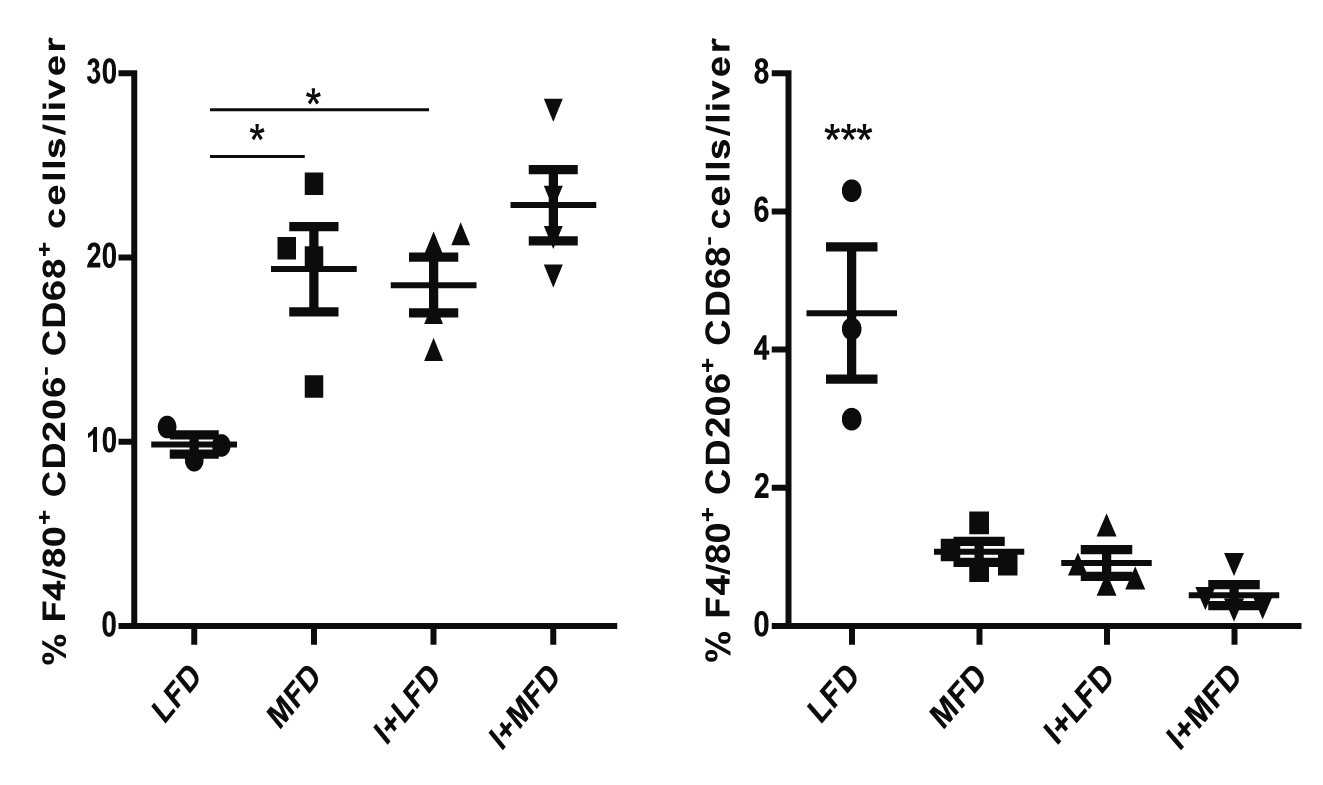

Supplement: S3 Fig — Percentages of hepatic F4/80+ cells of phenotype M1 (CD206-CD68+) and M2 (CD206+CD68-) are indicated. Data are shown as mean ± SEM of more than 3 mice per group from one experiment representative of three performed. (TIF) [file pntd.0003464.s003.tif]

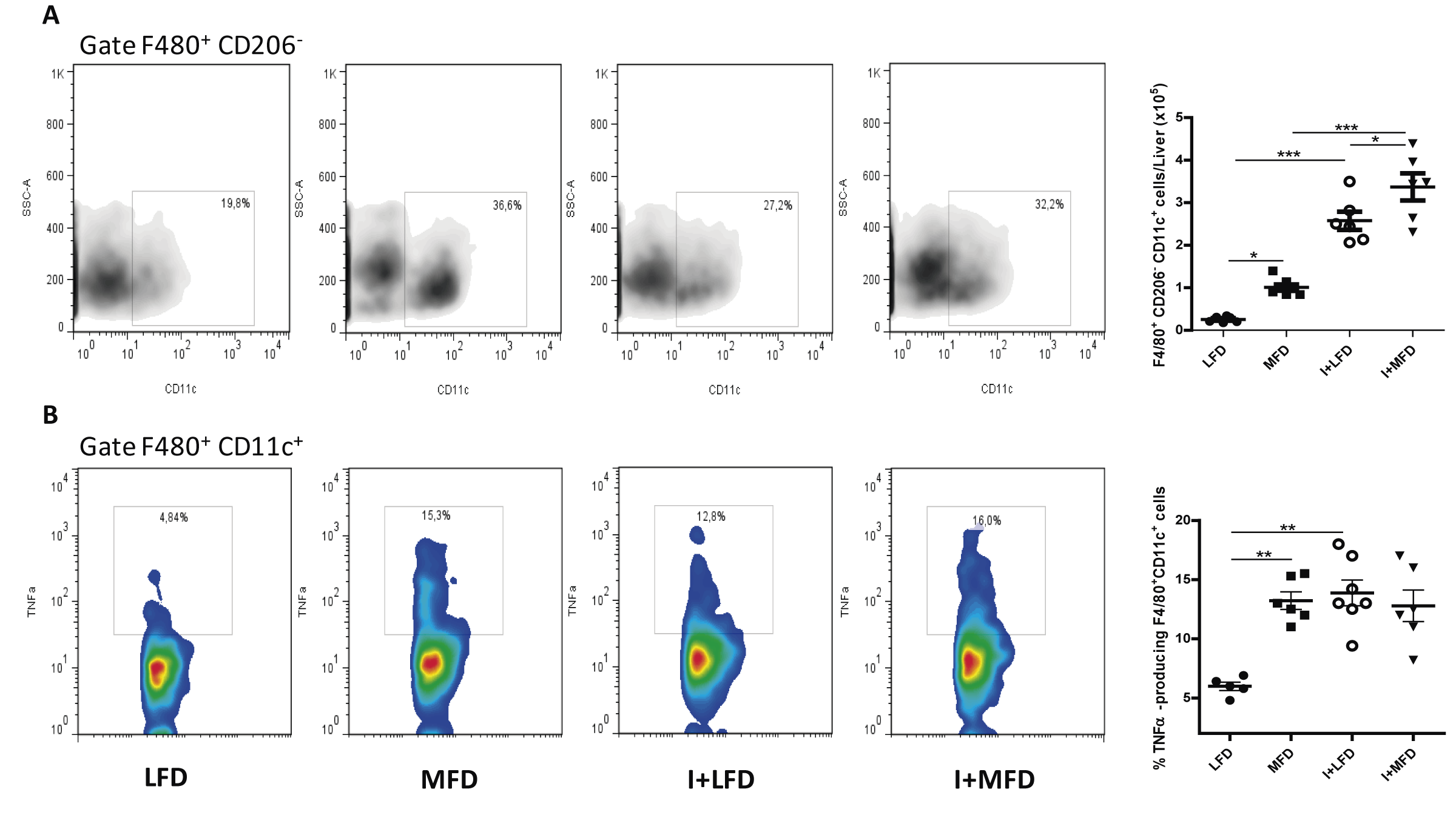

Supplement: S4 Fig — IHLs obtained at 24 weeks were stained with anti-F4/80, anti-CD11c, anti CD206 and anti-TNFα. (A) Percentage of F4/80+ CD206- CD11c+ cells of different groups of WT mice at 24 weeks is indicated. (B) IHLs from all groups were cultured in the presence of PMA plus ionomycin and brefeldin for 5 h and stained with anti-F4/80, anti-CD11c and anti-TNFα. A representative graphic is shown. Data are shown as mean ± SEM of more than 4 mice per group from one experiment representative of three performed. (TIF) [file pntd.0003464.s004.tif]

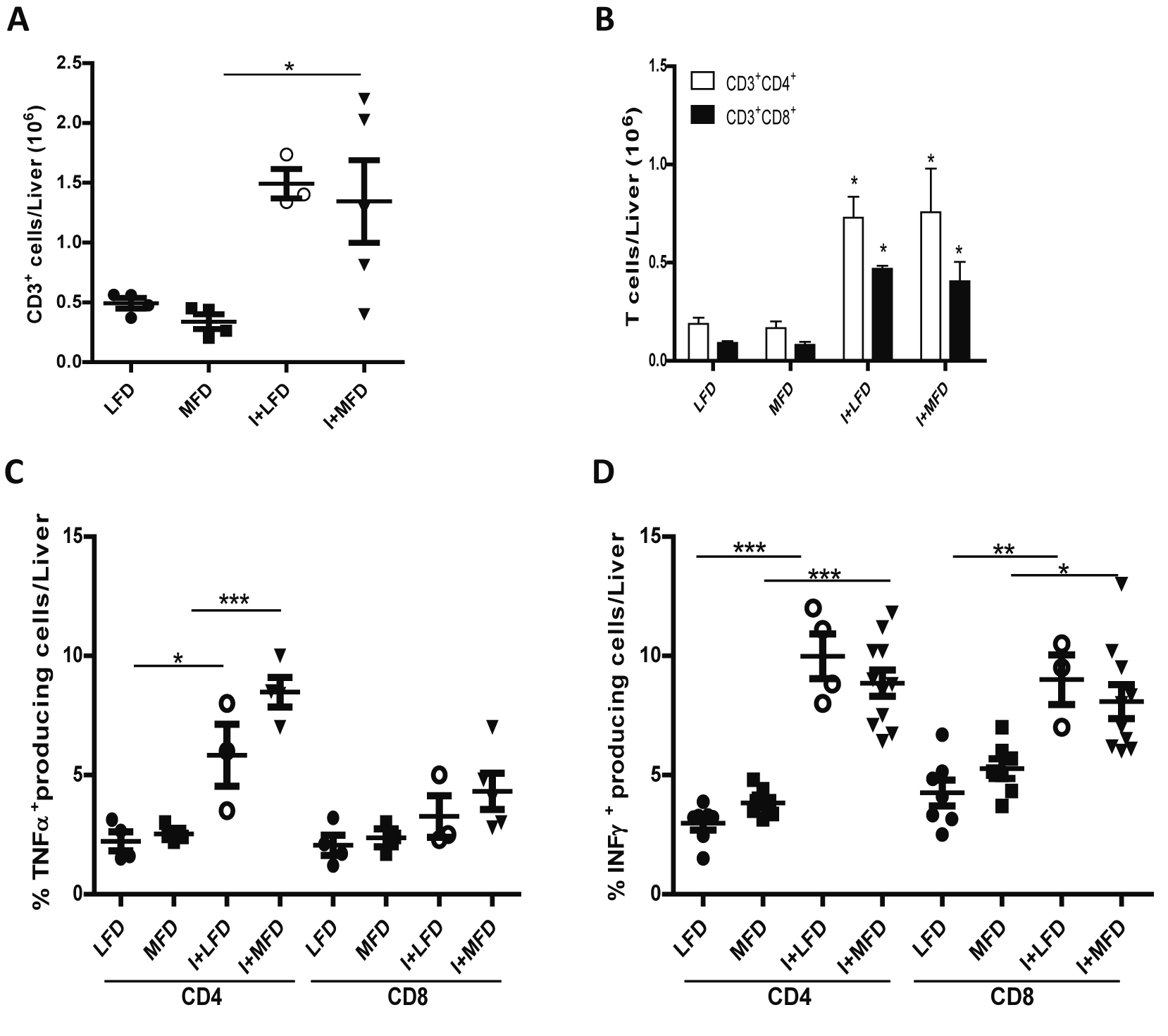

Supplement: S5 Fig — IHLs at 24 weeks were stained with anti-CD3, anti-CD4, anti-CD8, anti-TNFα and anti-IFNγ Abs. (A) The absolute numbers of CD3+ and (B) the absolute number of CD3+CD4+ and CD3+CD8+ cells in liver is indicated. (C) Percentage of TNFα and IFNγ producing CD4+ or CD8+ T cells in liver are shown by intracellular staining. IHLs from all groups were cultured in the presence of PMA plus ionomycin, monensin and brefeldin for 5 h and stained with corresponding antibodies. The results are expressed as mean ± SEM of more than 4 mice per group from one experiment representative of three performed. (TIF) [file pntd.0003464.s005.tif]

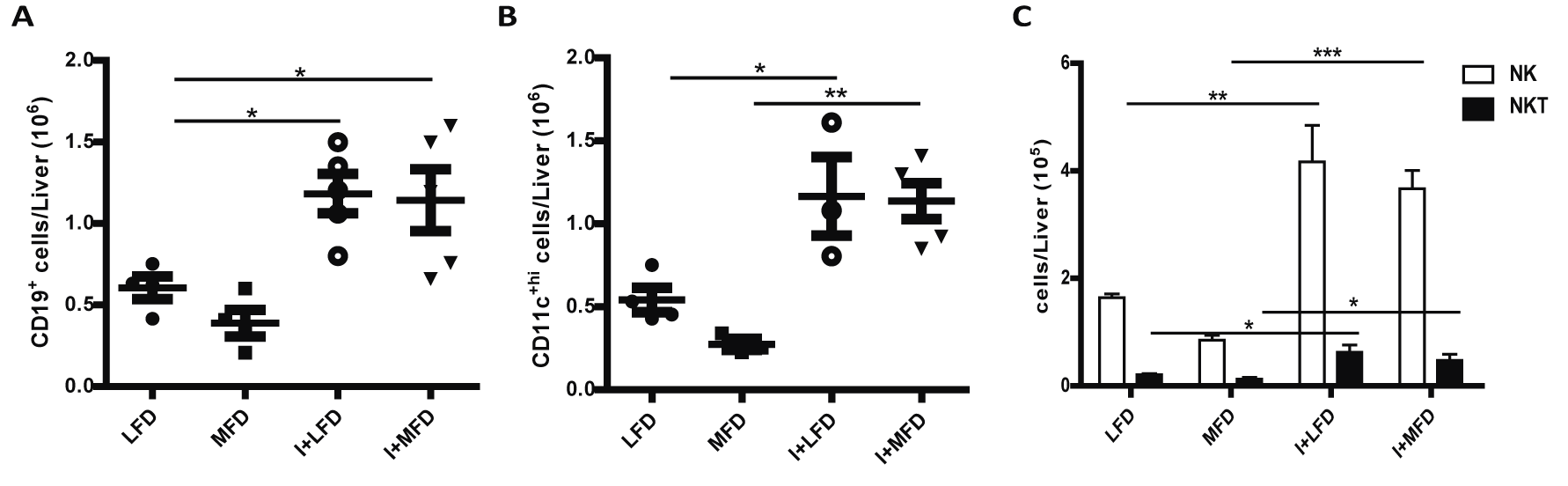

Supplement: S6 Fig — IHLs from different groups of mice were stained with anti-CD19, anti-CD11c, anti-CD3, anti-NK1.1. (A) The absolute numbers of CD19+, (B) the absolute number of CD11chi in liver and (C) the absolute number of NK and NKT cells are indicated. Data are shown as mean ± SEM of more than 3 mice per group from one experiment representative of three performed. (TIF) [file pntd.0003464.s006.tif]

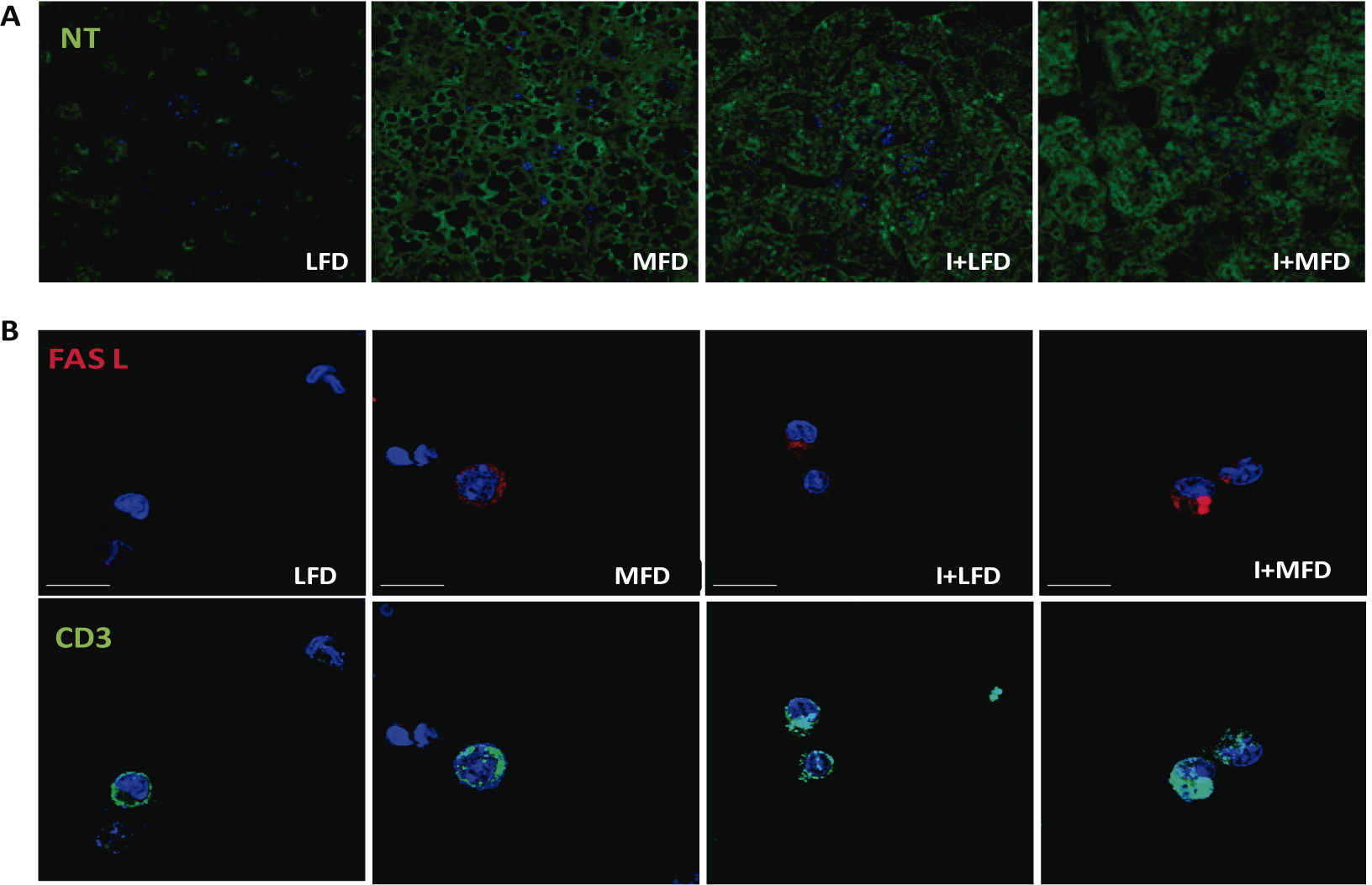

Supplement: S7 Fig — (A) 400x micrographs of hepatic tissue section allow panoramic evaluation of protein tirosine nitration after stained with Ab Alexa Fluor 488-anti- nitro tyrosine (NT). Photographs are representative of one out of five mice. (B) IHL were cultured with ConA for 48 h and labeled with FITC-anti mouse-CD3 and PE-anti FasL. DNA was stained with DNA-binding fluorochrome Hoechst 33258 (2 ug/mL). Slides were observed with a FV1000 (Olympus) confocal microscope. Scale bar: 10 μm. (TIF) [file pntd.0003464.s007.tif]
